# USING INNOVATIVE INTERGENERATIONAL STRATEGIES TO BOOST CAREERS IN AGING

**DOI:** 10.1093/geroni/igz038.2101

**Published:** 2019-11-08

**Authors:** Roma S Hanks

**Affiliations:** University of South Alabama, Mobile, Alabama, United States

## Abstract

Educational pipelines are effective in medical education with minority student populations. The University of South Alabama has a successful medical pipeline in the NIH/NIMHD-funded USA Center of Excellence – but no formal pipeline to support entry into careers in aging. In Gerontology and Geriatrics, the preponderance of pipelines focuses on students in advanced degree programs. The need remains largely unmet to inform young students about careers and research pathways in Gerontology before career and academic plans are established. The USA Gerontology Club initiated a student-led outreach to deliver information about careers in aging and academic programs in Gerontology and Geriatrics to high schools in communities with high health disparities. The program seeks to develop peer relationships with high school students to introduce them to careers in aging and related academic opportunities. The presentation includes barriers identified and development of a multi-phased, multi-disciplinary model leading to a formal pipeline for Gerontology.

